# Evaluation of automated anthropometrics produced by smartphone-based machine learning: a comparison with traditional anthropometric assessments

**DOI:** 10.1017/S0007114523000090

**Published:** 2023-09-28

**Authors:** Austin J. Graybeal, Caleb F. Brandner, Grant M. Tinsley

**Affiliations:** 1 School of Kinesiology & Nutrition, College of Education and Human Sciences, University of Southern Mississippi, Hattiesburg, MS 39406, USA; 2 Department of Kinesiology & Sport Management, Texas Tech University, Lubbock, TX 79409, USA

**Keywords:** Digital anthropometry, Mobile health, Body composition assessment, Obesity

## Abstract

Automated visual anthropometrics produced by mobile applications are accessible and cost effective with the potential to assess clinically relevant anthropometrics without a trained technician present. Thus, the aim of this study was to evaluate the precision and agreement of smartphone-based automated anthropometrics against reference tape measurements. Waist and hip circumference (WC; HC), waist:hip ratio (WHR) and waist:height ratio (W:HT) were collected from 115 participants (69 F) using a tape measure and two smartphone applications (MeThreeSixty^®^, myBVI^®^) across multiple smartphone types. Precision metrics were used to assess test-retest precision of the automated measures. Agreement between the circumferences produced by each mobile application and the reference were assessed using equivalence testing and other validity metrics. All mobile applications across smartphone types produced reliable estimates for each variable with intraclass correlation coefficients ≥ 0·93 (all *P* < 0·001) and root mean square coefficient of variation between 0·5 and 2·5 %. Precision error for WC and HC was between 0·5 and 1·9 cm. WC, HC, and W:HT estimates produced by each mobile application demonstrated equivalence with the reference tape measurements using 5 % equivalence regions. Mean differences via paired t-tests were significant for all variables across each mobile application (all *P* < 0·050) showing slight underestimation for WC and slight overestimation for HC which resulted in a lack of equivalence for WHR compared with the reference tape measure. Overall, the results of our study support the use of WC and HC estimates produced from automated mobile applications, but also demonstrates the importance of accurate automation for WC and HC estimates given their influence on other anthropometric assessments and clinical health markers.

Obesity is rapidly increasing with disparately high rates for individuals who are of low socio-economic status^(1)^ or living in rural communities^(2,3)^. Specifically, both low socio-economic status^(4,5)^ and rural occupancy^(6)^ are associated with higher rates of abdominal obesity, which is itself linked to a higher risk of cardiometabolic abnormalities^(7)^. Traditionally, abdominal obesity is evaluated by standard anthropometric assessments that include circumferences of the waist -and -hips specifically, given their association with cardiometabolic health risks^(8,9)^ and mortality^(10)^. Nevertheless, traditional anthropometric measures lack feasibility for those without access to clinical care, which is concerning given that there are few alternative methods that can successfully provide remote and cost-effective assessments without a trained technician present. Therefore, the development of remote healthcare tools that can provide accurate anthropometric assessments without additional costs are of critical importance.

Interestingly, the adaptations made to traditional healthcare models during the COVID-19 pandemic may have, unintentionally, provided a potential solution to this pressing issue. At the onset of the pandemic, healthcare facilities were forced to find remote alternatives to providing clinical care. Given that the majority of USA adults own a smartphone^(11)^ with a similar ownership existing across more vulnerable populations^(12,13)^, the demand for remote healthcare solutions led to the swift integration of mobile healthcare models that enable patients with access to care regardless of limited transportation or geographical location. In fact, recent evidence demonstrates the feasibility of mobile healthcare interventions in a rural setting showing reductions in waist circumference (WC) and visceral adiposity with this approach^(14)^. Additionally, and contrary to the observations made in a traditional healthcare setting^(15)^, intention to use and satisfaction with mobile health services is inversely associated with perceived health status which notes its utility for individuals of poor health. However, increased access to communication with healthcare providers is only one facet of the total healthcare experience. As such, the surge in mobile healthcare use coupled with the accelerated increase in obesity requires parallel advancements in remote health tools, such as automated anthropometrics, that can conveniently assess patient health status without additional costs.

Because virtually all smartphones can now employ high-resolution imaging through advances in smartphone camera technology, newly developed mobile applications can now leverage machine learning to automate anthropometric assessments from as little as two self-taken images. Furthermore, this method has recently shown to be associated with traditional measurement methods^(16)^, agree with multi-compartment body composition estimation models^(17)^ and demonstrates higher predictive ability of visceral adipose tissue compared with physical circumferences and other measures^(18)^. Thus, this methodological approach may be a potential solution to collecting large-scale anthropometric information and improve access to health information for those with monetary or geographic limitations. However, the consumer level and accessible nature of this method fosters continual industry competition and thus there are several mobile applications that claim to produce accurate automated anthropometric evaluations from smartphone-based imaging. As such, there are currently no studies, to our knowledge, that have fully assessed the equivalence of clinically significant measures of abdominal adiposity such as WC, hip circumference (HC), w:hip ratio (WHR) and waist:height ratio (W:HT) produced by multiple mobile applications across smartphone types to a reference measurement. Therefore, the purpose of this study was to determine the agreement and precision of automated anthropometric assessments produced by three-dimensional (3D) mobile scanning applications compared with a reference tape measure.

## Methods

### Participants

A total of 115 individuals (F: 69, M: 46) between ages 18 and 75 years were prospectively recruited for this cross-sectional study. Participants were excluded if they were younger than 18 or older than 75; were missing any limbs or part of a limb that influenced an accurate assessment of the primary anthropometric measures; were pregnant; trying to become pregnant or breast-feeding or lactating. The study took place from March 2022 through July 2022 and was conducted according to the guidelines laid down in the Declaration of Helsinki, with all procedures involving human participants approved by the university ethics committee (IRB#21–213). Written informed consent was obtained from all participants.

### Procedures

Our procedures for visual body composition scanning have been previously reported^(19)^ but are summarised below. Participants reported to the laboratory after abstention from food, beverages, supplements/medication and exercise for ≥ 8 h. Upon arrival participants were asked to remove any external accessories (jewelry, shoes, etc.) and/or loose clothing and underwent measurements of height collected by a digital stadiometer (SECA, Hamburg, Germany), weight collected by a calibrated digital scale (SECA, Hamburg, Germany) and WC and HC collected using an aluminum tape measure. Following tape measurements, participants were lead to a specific area of the laboratory to complete the smartphone-based assessments. For scanning on each mobile application, participants were instructed to wear minimal form-fitting clothing. For example, female participants were instructed to wear a sports bra and tight-fitting shorts/leggings, and male participants were instructed to wear compression shorts/tights only. Higher waisted shorts that covered the participants bellybutton were altered to expose the participants entire abdominal region to the smartphone camera. Participants with long hair were instructed to tie their hair up so that no hair was present below the shoulder line.

### Reference tape measurements

Traditional WC and HC were collected by an aluminum tape measure, and these measurements were used as, or used to calculate, all reference variables for this study. Because there are no standardised measurement sites for WC and HC across mobile applications, estimates of WC and HC from these applications could be generated from different locations at or around the waist and hip regions. Therefore, the reference tape measurements were standardised to specific locations, where the reference WC was measured at the level of the iliac crest^(20)^ and the reference HC was measured at the widest portion of the lateral hips^(9)^ given the ease in the visual detection of pronounced or distinct body areas during the landmarking procedures of smartphone-based imaging. In addition, the American Heart Association waist circumference risk classifications are based off measurements collected at the level of the iliac crest^(21)^. All tape measurements were conducted by the same two investigators for all participants. WC and HC were used to produce a WHR by dividing WC by HC and measurements of W:HT by dividing WC by height collected from the digital stadiometer. For WHR measurements, the first WC was divided by the first HC and the second WC by the second HC. Because height was measured at a single timepoint, both the first and second WC were divided by the same height measurement. All measurements were conducted in duplicate and averaged to produce a final estimate.

### Mobile applications and smartphone types

Our procedures for each smartphone application and type have been described elsewhere^(19)^. Two mobile applications were used for this study which included MeThreeSixty^®^ (ME360; Size Stream LLC) and myBVI^®^ (Select Research LTD). Because mobile applications are frequently updated under real-world circumstances, and because these updates are often necessary to fix unavoidable issues with the application’s performance, applications were updated daily prior to testing, when available. The study began with software version 3.3.0 (ME360_Apple)_, 3.2.2 (ME360_Samsung)_ and 3.0.0 (myBVI^®^) and ended using versions 3.4.2 (both ME360) and 3.1.2 (myBVI^®^), respectively. Further, two different smartphones were used for this study to compare the precision and agreement of the applications across smartphone types. The smartphones used in this study included an iPhone^®^ 12 Pro (Apple^®^ Inc.) and a Samsung Galaxy^®^ S21+ (Samsung^®^ Group). Assessments using the iPhone^®^ were conducted using the same software version for the entirety of the study (iOS 15·0·1) but due to Samsung’s^®^ forced security updates, multiple software versions were employed for this smartphone specifically (One UI version 3·1, 4·0 and 4·1 and Android^®^ version 11 and 12). All images for ME360 were collected using the front facing camera from both smartphones, whereas images from myBVI^®^ were collected using the front facing camera from the iPhone^®^ only due to compatibility issues between myBVI^®^ and Samsung Galaxy^®^.

### Anthropometric assessment protocol for the mobile applications

Our procedures for collecting smartphone-based body composition estimates have been previously reported^(19)^. To perform the assessments for each mobile application, participants were taken to a designated area of the laboratory that did not have any objects or light behind the participants’ back. All images were taken in front of a gray vinyl wall in this designated area, and all external windows were covered so that no other background or external light source polluted the scanning region. The smartphone was positioned at a standardised distance from the participant’s mid-foot unless instructed otherwise by the application and a standardised height for all participants using a stationary tripod with adjustable angle settings. The smartphone order was assigned randomly for each participant. Each participant’s personal information (age, sex, height and weight) was uploaded into the application before testing commenced. The smartphone was locked into place at an angle determined appropriate by the mobile application. Once the smartphone and the participant were situated appropriately, participants were asked to stand in two positions, in accordance with the manufacturer guidelines, while images were collected. For the first image, participants faced the camera and stood with arms and feet positioned away from the torso. For the second image, participants were instructed to turn to their profile with either their left (ME360) or right (myBVI^®^) shoulder facing the camera, face forward, extend the elbows completely and place their hands against their lateral thigh while images were collected. All assessments were conducted in duplicate and subjectively inspected for quality to ensure that there were no errors during landmarking procedures. WC and HC from ME360 were provided directly by the mobile application. WHR_ME360_ was calculated as WC_ME360_ divided by HC_ME360_. Because myBVI^®^ provides WHR and W:HT, but not WC or HC, WC_myBVI_ was calculated as height from the stadiometer multiplied by W:HT_myBVI_. The newly produced WC_myBVI_ was divided by WHR_myBVI_ to produce HC_myBVI_. Because myBVI^®^ requires the user to round their height, and because ME360_W:HT_ used height measured by stadiometer, we calculated W:HT_myBVI_ by dividing WC_myBVI_ from the stadiometer height. For WHR measurements from each application, the first automated WC was divided by the first automated HC and the same was done for the second scans. Similar to the reference method, both the first and second automated WC were divided by the same height measurement. All measurements were conducted in duplicate and averaged to produce a final estimate.

### Statistical analysis

We conducted a non-directional power analysis to determine the sample size necessary to detect significant differences using a paired-samples *t* test (the primary statistical analysis for determining group mean differences (MD)). Prior to analysis, we determined ±4·0 cm to be a meaningful MD and thus using a MD of 4·0 ± 6·0 and an α = 0·05 it was determined that twenty participants were necessary to observe at least 80 % power. All outcome variables were normally distributed as assessed by Shapiro–Wilk and visual inspection of Q-Q plots. Means and 95 % confidence intervals (95 %CI) for each device were calculated for WC, HC, WHR and W:HTand the MD and 95 %CI for each variable were calculated as the mobile application in question minus the reference. Test-retest precision of each anthropometric assessment was assessed using intraclass correlation coefficients (ICC) with two-way, random effects and absolute agreement. Precision was also measured using precision error (PE) and root mean square coefficient of variation (RMS-%CV). For ME360, precision metrics were used to determine precision between smartphone types using the first scan from each smartphone. Because WHR and W:HT are unitless, PE was not calculated for these variables. A device error resulted in one participant (*n* 1) missing a single scan from one smartphone (Samsung^®^) for ME360. Therefore, 114 participants were used to determine precision, and 115 participants were used to assess agreement. An average of the two scans for each mobile application was used to determine agreement, and only one scan was used for the participant with a missing scan. Equivalence testing was used to determine equivalence with the reference tape measurements for WC, HC, WHR and W:HT using 5 % equivalence regions. Additionally, and because WC is often used to assess abdominal obesity for the evaluation of cardiometabolic health risk, the percentage of correct abdominal obesity classifications according to the guidelines put forth by the American Heart Association (≥ 88 cm for females; ≥ 102 cm for males) are presented for each mobile assessment^(21)^. Agreement with the reference method was also assessed by separate paired samples *t* test, Pearson correlation coefficients, root mean square error (RMSE = √∑(predicted-actual)^2^/n) and standard error of the estimate. Individual accuracy was assessed using the methods of Bland and Altman^(22)^ to determine the 95 % limits of agreement (LOA), and regression techniques were used to determine proportional biases. Subgroup analyses were conducted for sex and racial differences. For race, analyses were conducted for non-Hispanic white and non-Hispanic Black/African-American (B/AA) individuals (Table 1). Data from other racial and ethnic groups were included in the complete sample analyses only. Anthropometric differences between groups were assessed by independent samples *t* test. Statistical significance was accepted at *P* < 0·05. Data were analysed using the TOSTER package^(23)^ in R version 4.1.2, IBM SPSS version 27 and Microsoft Excel version 16.

**Table 1.** Participant characteristics

		Total		Male		Female	
*n* 115		*n*	%	*n*	%	*n*	%
Sex				46	40·0	69	60·0
Race	White	83	72·2	31	67·4	52	75·4
	Black/AA	28	24·3	14	30·4	14	20·3
	Asian	4	3·5	1	2·2	3	4·3
Ethnicity	Hispanic	7	6·1	2	4·3	5	7·2
		Mean	95 % CI	Mean	95 % CI	Mean	95 % CI
Anthropometry	Age (year)	29·4	27·1, 31·7	26·5	23·8, 29·1	31·3	28·0, 34·6‡
	Height (cm)	170·2	168·4, 172·0	178·0	175·5, 180·4	165·0	163·5, 166·5*
	Weight (kg)	80·4	76·3, 84·4	94·3	87·4, 101·1	71·07	67·5, 74·7*
	BMI (kg/m^2^)	27·5	26·2, 28·7	30·2	27·9, 32·5	25·6	24·4, 26·9†
	Waist (cm)	91·4	88·5, 94·3	96·2	91·2, 101·3	88·2	84·9, 91·6†
	Hip (cm)	103·0	101·7, 106·1	106·2	102·3, 110·5	102·4	99·8, 105·0
	Waist:Hip	0·88	0·86, 0·89	0·90	0·89, 0·92	0·86	0·84, 0·88†
	Waist:Height	0·54	0·52, 0·56	0·54	0·51, 0·57	0·54	0·51, 0·57
		*n*	%	*n*	%	*n*	%
BMI Classification	≥ 40 kg/m^2^	5	4·3	4	8·7	1	1·4
	30–39·9 kg/m^2^	23	20·0	11	23·9	12	17·4
	25–29·9 kg/m^2^	39	33·9	20	43·5	19	27·5
	< 25 kg/m^2^	48	41·7	11	23·9	37	53·6
*n* 104		White (*n* 76)		Black (*n* 28)			
		Mean	95 % CI	Mean	95 % CI		
Anthropometrics	Height (cm)	170·5	168·4, 172·6	171·2	167·0, 175·5		
	Weight (kg)	77·1	73·4, 80·8	94·1	82·0, 106·1‡		
	BMI (kg/m^2^)	26·3	25·2, 27·4	31·8	28·0, 35·7†		
	Waist (cm)	89·4	86·6, 92·3	99·9	91·2, 108·6‡		
	Hip (cm)	102·5	100·3, 104·7	109·5	103·1, 116·1‡		
	Waist:Hip	0·87	0·86, 0·89	0·91	0·87, 0·94‡		
	Waist:Height	0·53	0·51, 0·54	0·59	0·53, 0·64‡		
		*n*	%	*n*	%		
BMI Classification	≥ 40 kg/m^2^	0	0·0	5	17·9		
	30–39·9 kg/m^2^	14	18·4	9	32·1		
	25–29·9 kg/m^2^	31	40·8	4	14·3		
	< 25 kg/m^2^	31	40·8	10	35·7		

AA, African-American.

* Statistically significant at *P* < 0·001.

† Statistically significant at *P* < 0·010.

‡ Statistically significant at *P* < 0·050.

## Results

Participant characteristics are reported as mean and the 95 %CI or as a percentage of the total for each column (Table 1).

### Precision analysis

Results of the precision analysis are presented in Table 2. For precision, all ICC, including assessments between smartphone types, ranged from 0·933 to 0·998 (all *P* < 0·001). For WC and HC, ME360_Apple_ produced the lowest PE and RMS-%CV followed by tape measurements and ME360_Samsung_. myBVI^®^ had the largest PE and RMS-%CV for both WC and HC across applications. Overall, precision was lower between smartphone types for WC and HC produced by ME360_Apple × Samsung_. RMS-%CV for WHR was lowest for ME360_Apple_ (0·70 %) which was slightly lower than ME360_Samsung_ (0·80 %), but both ME360_WHR_ estimates had lower RMS-%CV than those conducted by tape measurement (1·05 %). myBVI^®^ had the highest RMS-%CV for WHR (2·43 %) which was also higher than the RMS-%CV between smartphone types for ME360_Apple × Samsung_ (1·98 %).

**Table 2.** Precision analysis of smartphone-based automated anthropometrics

*n* 114		ICC*	RMS-%CV	PE†
WC	Tape Measure	0·998	0·78	0·63
	ME360_Apple_	0·998	0·69	0·54
	ME360_Samsung_	0·996	1·11	0·87
	myBVI	0·995	1·43	1·12
	ME360_Apple × Samsung_	0·979	2·54	1·91
HC	Tape Measure	0·997	0·70	0·65
	ME360_Apple_	0·998	0·54	0·51
	ME360_Samsung_	0·994	0·95	0·90
	myBVI	0·982	2·07	1·91
	ME360_Apple × Samsung_	0·979	1·72	1·59
WHR	Tape Measure	0·984	1·05	
	ME360_Apple_	0·992	0·70	
	ME360_Samsung_	0·991	0·80	
	myBVI	0·933	2·43	
	ME360_Apple × Samsung_	0·952	1·98	
W:HT	Tape Measure/Stadiometer	0·997	0·79	
	ME360_Apple_	0·997	0·71	
	ME360_Samsung_	0·994	1·12	
	myBVI	0·995	1·42	
	ME360_Apple × Samsung_	0·978	2·51	

HC, hip circumference; ICC, intraclass correlation coefficient (two-way random effects, absolute agreement and single measurement); PE, precision error; RMS-%CV, root mean square coefficient of variation (%); WC, waist circumference; WHR, waist:hip ratio; W:HT, waist:height ratio.

* *P* < 0·001 for all measurements.

† Measurements are in cm.

### Agreement analysis

Results of the agreement analysis for the total sample are presented in Table 3. Overall, the number of correct abdominal obesity classifications for each mobile application was 103 (89·6 %) for ME360_Apple_, 104 (90·4 %) for ME360_Samsung_ and 106 (92·2 %) for myBVI^®^. Of the incorrect classifications, both ME360_Apple_ (twelve incorrect) and myBVI^®^ (nine incorrect) incorrectly classified participants as having abdominal obesity on three occasions with the remaining incorrectly classifying participants as not having abdominal obesity. ME360_Samsung_ incorrectly classified participants (eleven incorrect) as having abdominal obesity on five occasions with the remaining incorrectly classifying participants as having abdominal obesity.

**Table 3.** Agreement between smartphone-based automated anthropometrics and reference tape measurements

*n* 115		Mean	95 %CI*	MD	95 %CI*	EQ	RMSE	95 % LOA	SEE	*β*	R^2^
WC	Tape Measure	91·4	88·5, 94·4								
	ME360_Apple_	89·1	86·6, 91·7	–2·3	–3·4, −1·2†	Y	6·5	11·9	2·4	–0·15†	0·86
	ME360_Samsung_	89·9	87·4, 92·4	–1·6	–2·7, −0·4§	Y	6·5	12·4	2·6	–0·15†	0·84
	myBVI	88·9	85·9, 91·9	–2·5	–3·4, −1·7†	Y	5·2	9·0	1·5	0·03	0·92
HC	Tape Measure	103·9	101·7, 106·1								
	ME360_Apple_	107·0	105·0, 109·1	3·1	2·5, 3·8†	Y	4·7	6·8	1·4	–0·07§	0·91
	ME360_Samsung_	107·6	105·5, 109·7	3·7	3·1, 4·4†	Y	5·1	6·7	1·5	–0·06§	0·92
	myBVI	105·8	103·1, 108·4	1·9	0·87, 2·9†	Y	5·8	10·7	2·1	0·20†	0·87
WHR	Tape Measure	0·88	0·86, 0·89								
	ME360_Apple_	0·83	0·82, 0·84	–0·05	–0·06, −0·04†	N	0·07	0·11	0·05	–0·06	0·48
	ME360_Samsung_	0·83	0·82, 0·85	–0·04	–0·05, −0·03†	N	0·07	0·11	0·05	–0·08	0·47
	myBVI	0·84	0·83, 0·85	–0·04	–0·05, −0·03†	N	0·06	0·09	0·03	–0·07	0·66
W:HT	Tape Measure/Stadiometer	0·54	0·52, 0·56								
	ME360_Apple_	0·52	0·51, 0·54	–0·014	–0·021, −0·008†	Y	0·04	0·07	0·01	–0·17§	0·86
	ME360_Samsung_	0·53	0·51, 0·54	–0·009	–0·016, −0·002‡	Y	0·04	0·07	0·02	–0·18§	0·85
	myBVI	0·52	0·51, 0·54	–0·015	–0·020, −0·010†	Y	0·03	0·05	0·01	0·01	0·92

*β*, regression coefficient produced from linear regression as used to assess proportional bias; EQ, equivalence; HC, hip circumference; LOA, limits of agreement; MD, mean difference; RMSE, root mean square error; SEE, standard error of the estimate; WC, waist circumference; WHR, waist:hip ratio; W:HT, waist:height ratio.

* Expressed as mean (95 % CI).

† Indicates statistical significance at *P* < 0·001.

‡ Indicates statistical significance at *P* < 0·010.

§ Indicates statistical significance at *P* < 0·05.

**Fig. 1.**
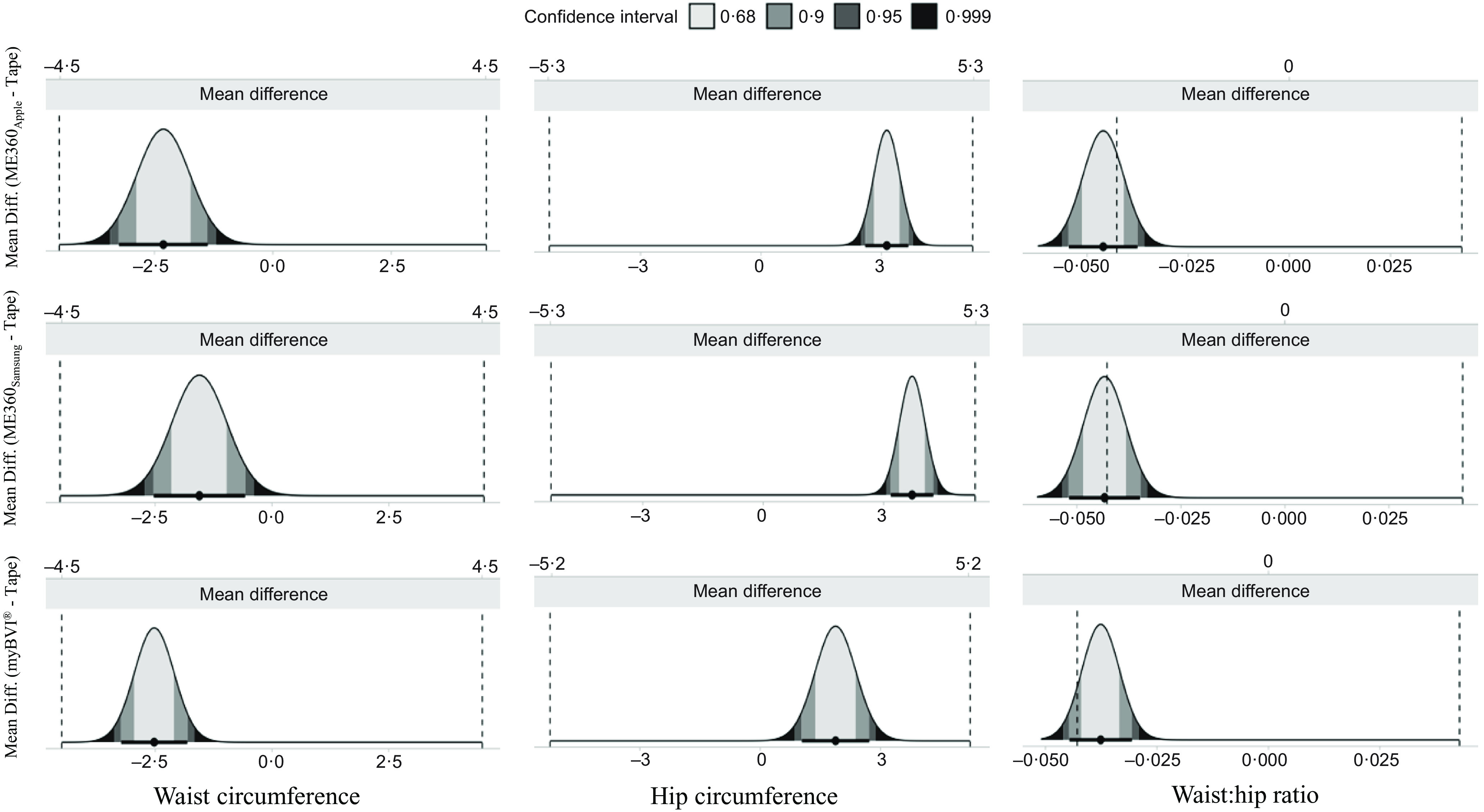
Equivalence between smartphone-based automated anthropometrics and reference tape measurements. An illustration of the equivalence between waist circumference (WC), hip circumference (HC) and waist:hip ratio (WHR) produced by each mobile application and those produced by the reference is shown. Variables are considered equivalent with the tape measurements when the entire 90 %CI is within the equivalence region.

Paired *t* tests revealed that all variables produced by the mobile applications differed significantly from the reference tape measurement for the total sample. WC was slightly underestimated by each mobile application (all MD: ≤ −2·5 cm, *P* < 0·050) relative to the reference method for each application. Conversely, HC was slightly overestimated across each mobile application relative to the reference method with myBVI^®^ demonstrating the lowest MD (1·9 cm) followed by ME360 with negligible differences between smartphone types (all *P* < 0·050). MD for WHR and W:HT were similar across devices. Despite significantly different MD, equivalence testing revealed that all devices demonstrated equivalence to the reference method using a 5 % equivalence region for WC, HC and W:HT (Fig. 1, Table 3). The slight underestimation of WC and the slight overestimation of HC resulted in a non-significant equivalence test for WHR. ME360 demonstrated the highest RMSE for WC (±6·5 cm for each) but the lowest RMSE for HC (±4·7 to ±5·1 cm). RMSE for WHR and W:HT was similar across applications.

**Fig. 2.**
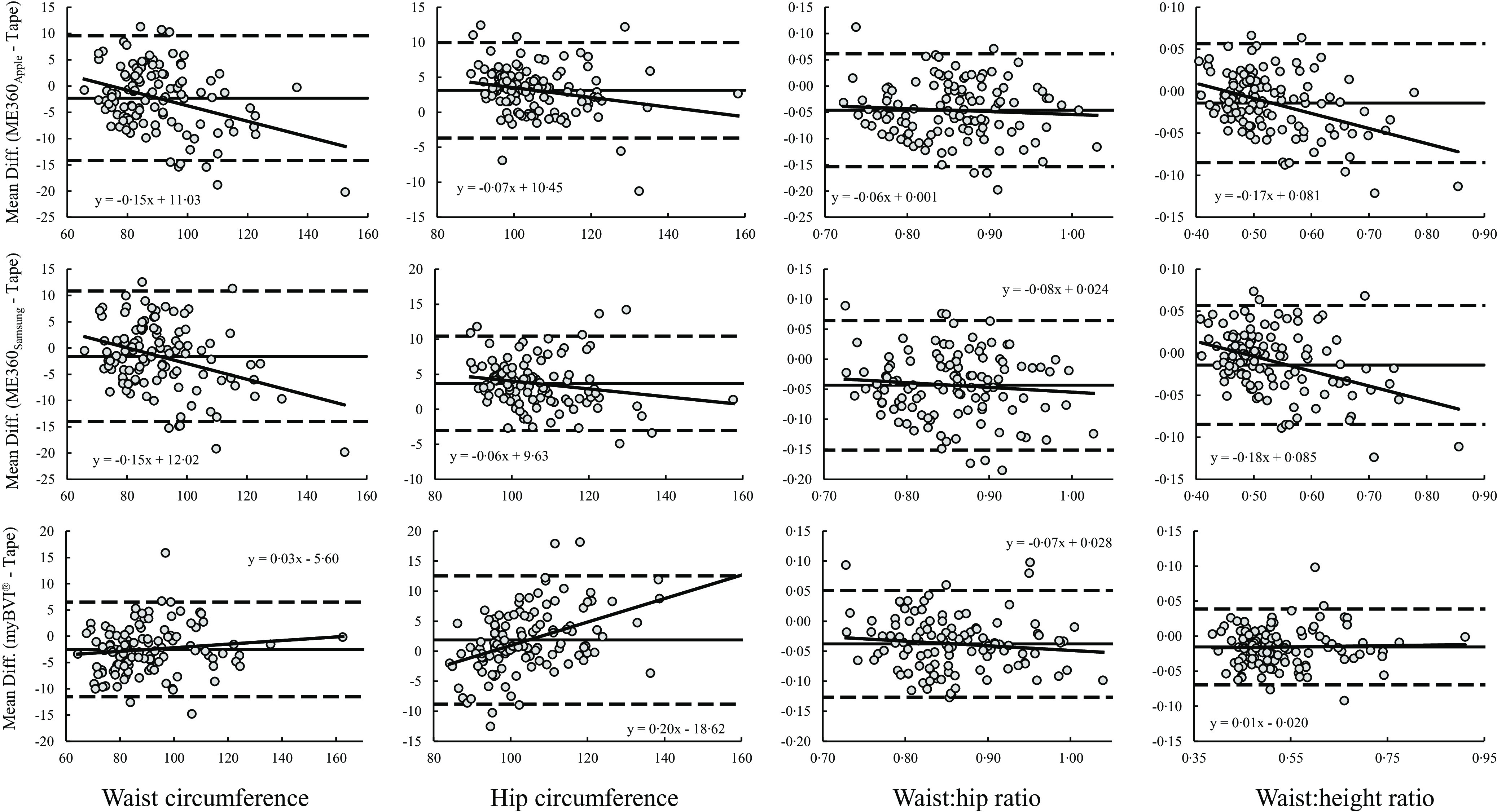
Bland–Altman plots of smartphone-based automated anthropometrics. Bland–Altman plots are presented. Solid diagonal line: relationship between the mean difference in circumference estimates (y-axis) and the average of the automated and tape measurements (x-axis). Solid horizontal line: average mean difference. Dashed horizontal lines: 95 % limits of agreement.

Results for the Bland–Altman analysis for the total sample are displayed in Table 3 and illustrated in Fig. 2. LOA ranged from ±9·0 to ±12·4 cm for WC and from ±6·7 to ±10·7 cm for HC. For WHR and W:HT, LOA were similar across applications. Significant proportional bias was observed for WC estimates produced by ME360 but not myBVI^®^. Conversely, significant proportional bias was observed for HC estimates produced by all methods.

### Sex differences in agreement

Agreement analyses by sex groups are presented in Table 4. When stratified by sex, there were no significant differences between the WC estimates produced by each device and the reference method for males (all *P* > 0·050); however, WC was significantly underestimated for females across all devices (all *P* < 0·001). Significant overestimations of HC were observed for both males and females using ME360 (*P* < 0·001) but only for males using myBVI^®^ (*P* < 0·001). WHR was significantly underestimated for both males and females across all applications (all *P* < 0·05). For WC, RMSE was lower for males across all devices. RMSE for HC estimates was negligible between males and females using ME360 but were substantially higher for males using myBVI^®^. RMSE for WHR was similar between males and females other than WHR estimates produced by ME360_Apple_ which, for females, was more than double that of males.

**Table 4.** Agreement between smartphone-based automated anthropometrics and reference tape measurements by sex groups

		Males (*n* 46)						Females (*n* 69)					
*n* 115		MD	95 %CI*	RMSE	LOA	*β*	R^2^	MD	95 %CI	RMSE	LOA	*β*	R^2^
WC	ME360_Apple_	–0·3	–1·7, 1·2	4·8	9·6	–0·20†	0·94	–3·7	–5·2, −2·1†	7·4	12·6	–0·22†	0·80
	ME360_Samsung_	0·6	–1·0, 2·2	5·3	10·4	–0·22†	0·93	–3·0	–4·6, −1·4†	7·2	12·9	–0·21†	0·78
	myBVI	–0·4	–1·3, 0·6	3·3	6·6	–0·01	0·93	–4·0	–5·1, −2·8†	6·2	9·3	0·01	0·89
HC	ME360_Apple_	2·6	1·5, 3·7†	4·6	7·2	–0·02	0·92	3·5	2·7, 4·3†	4·8	6·5	–0·11‡	0·91
	ME360_Samsung_	3·5	2·4, 4·5†	5·1	6·8	–0·03	0·93	3·9	3·1, 4·7†	5·2	6·7	–0·08	0·90
	myBVI	5·9	4·4, 7·4†	6·0	10·1	0·14§	0·89	–0·8	–1·7, 0·1	3·8	7·4	0·13‡	0·91
W:Hip	ME360_Apple_	–0·02	–0·03, −0·01‡	0·03	0·08	–0·30§	0·51	–0·06	–0·08, −0·05†	0·07	0·11	–0·25§	0·44
	ME360_Samsung_	–0·02	–0·03, −0·01‡	0·07	0·11	–0·35‡	0·58	–0·06	–0·07, −0·04†	0·06	0·12	–0·26§	0·40
	myBVI	–0·05	–0·06, −0·03†	0·06	0·08	0·07	0·60	–0·03	–0·04, −0·02†	0·04	0·09	–0·10	0·62
W:Height	ME360_Apple_	–0·00	–0·01, 0·01	0·03	0·05	–0·17	0·95	–0·02	–0·03, −0·01†	0·04	0·08	–0·20‡	0·79
	ME360_Samsung_	0·00	–0·01, 0·01	0·03	0·06	–0·20†	0·94	–0·02	–0·03, −0·01‡	0·04	0·08	–0·20‡	0·78
	myBVI	–0·00	–0·01, 0·01	0·02	0·04	–0·02†	0·97	–0·02	–0·03, −0·02†	0·04	0·06	0·01	0·88

*β*, regression coefficient produced from linear regression as used to assess proportional bias; EQ, equivalence; HC, hip circumference; LOA, limits of agreement; MD, mean difference; R^2^, r-squared; RMSE, root mean square error; SEE, standard error of the estimate; WC, waist circumference; WHR, waist:hip ratio; W:HT, waist:height ratio.

* Expressed as mean (95 % CI).

† Indicates statistical significance at *P* < 0·001.

‡ Indicates statistical significance at *P* < 0·010.

§ Indicates statistical significance at *P* < 0·05.

Results for the Bland–Altman analysis by sex group are displayed in Table 4. LOA ranged from ±6·6 to ±12·9 cm for WC and were smaller in males across all devices. LOA ranged from ±6·5 to ±10·1 cm for HC and were smaller in females across all devices, albeit similar for ME360. For WHR and W:HT, LOA were similar between males and females although all LOA were slightly lower for males. Significant proportional biases were observed for WC estimates produced by ME360 (all *P* < 0·001), but not myBVI® (both *P* > 0·050) and were similar between males and females. Significant proportional biases were observed for HC estimates produced by myBVI^®^ and were similar between males and females (both *P* < 0·050). For HC produced by ME360, no proportional bias was observed for males, but was observed for females using ME360_Apple_ (*P* < 0·010) but not ME360_Samsung_ (*P* = 0·058). Significant proportional biases were observed for WHR produced by ME360 in both males and females (all *P* < 0·050) but not for WHR produced by myBVI^®^ (*P* < 0·050).

### Racial differences in agreement

Agreement analyses by the race groups evaluated in this study are presented in Table 5. WC estimates from ME360_Apple_, but not ME360_Samsung_, were significantly underestimated for both White and B/AA participants (*P* < 0·010). Although non-significant, the MD for WC produced by ME360_Samsung_ for B/AA participants was more than double the MD for White participants. myBVI^®^ significantly underestimated WC for White participants (*P* < 0·001) but not B/AA participants (*P* = 0·08). HC estimates from each application were all significantly overestimated (all *P* < 0·050); however, MD for HC estimates were markedly higher for B/AA participants compared with White participants. WHR estimates were significantly underestimated for all applications (all *P* < 0·001) and were similar between White and B/AA participants. For WC, RMSE was similar between White and B/AA participants across devices; however, RMSE for HC was substantially higher for B/AA participants across applications. RMSE for WHR and W:HT was similar between White and B/AA participants across applications.

**Table 5.** Agreement between smartphone-based automated anthropometrics and reference tape measurements between White and Black/African American participants

		White (*n* 76)						Black/African American (*n* 28)					
		MD	95 %CI*	RMSE	LOA	*β*	R^2^	MD	95 %CI	RMSE	LOA	*β*	R^2^
WC	ME360_Apple_	–1·8	–3·2, −0·5‡	6·1	11·5	–0·14§	0·77	–3·5	–6·1, −0·9§	7·4	13·1	–0·18‡	0·93
	ME360_Samsung_	–1·0	–2·4, 0·4	6·3	12·2	–0·15§	0·75	–2·5	–5·2, 0·1	7·2	13·4	–0·18‡	0·92
	myBVI	–2·7	–3·7, −1·6†	5·3	9·0	0·01	0·87	–1·6	–3·4, 0·2	4·9	9·2	0·02	0·96
HC	ME360_Apple_	2·9	2·2, 3·5†	4·1	5·9	–0·11‡	0·90	4·2	2·4, 6·0†	6·2	9·1	–0·09	0·93
	ME360_Samsung_	3·3	2·6, 3·9†	4·3	5·5	–0·11‡	0·92	5·6	3·8, 7·3†	7·0	8·7	–0·09	0·93
	myBVI	1·3	0·2, 2·4§	4·9	9·2	0·18†	0·83	4·8	2·5, 7·2†	7·7	11·9	0·14§	0·91
WHR	ME360_Apple_	–0·04	–0·05, −0·03†	0·07	0·10	0·00	0·50	–0·06	–0·08, −0·04†	0·08	0·11	–0·19	0·55
	ME360_Samsung_	–0·04	–0·05, −0·02†	0·07	0·11	–0·00	0·48	–0·06	–0·08, −0·04†	0·08	0·10	–0·26	0·58
	myBVI	–0·04	–0·05, −0·03†	0·06	0·09	0·06	0·64	–0·05	–0·07, −0·04†	0·06	0·07	0·01	0·82
W:HT	ME360_Apple_	–0·01	–0·02, −0·00§	0·04	0·07	–0·15§	0·78	–0·02	–0·04, −0·01‡	0·04	0·08	–0·20‡	0·94
	ME360_Samsung_	–0·00	–0·01, 0·00	0·04	0·08	–0·19‡	0·75	–0·02	–0·03, −0·00	0·04	0·07	–0·20‡	0·92
	myBVI	–0·01	–0·02, −0·00†	0·03	0·06	–0·03	0·86	–0·01	–0·02, −0·00	0·03	0·05	0·01	0·95

*β*, regression coefficient produced from linear regression as used to assess proportional bias; EQ, equivalence; HC, hip circumference; LOA, limits of agreement; MD, mean difference; R^2^, r-squared; RMSE, root mean square error; SEE, standard error of the estimate; WC, waist circumference; WHR, waist:hip ratio; W:HT: waist:height ratio.

* Expressed as mean (95 % CI).

† Indicates statistical significance at *P* < 0·001.

‡ Indicates statistical significance at *P* < 0·010.

§ Indicates statistical significance at *P* < 0·05.

Results for the Bland–Altman analysis by race are displayed in Table 5. LOA ranged from ±9·0 to ±13·4 cm for WC and were similar for White and B/AA participants. For HC, LOA ranged from ±5·5 to ±11·9 and were noticeably higher for B/AA participants for each device. For WHR and W:HT, LOA were similar for White and B/AA participants. Significant proportional bias was observed for WC and W:HT estimates produced by ME360 (all *P* < 0·050) but not myBVI^®^ which were similar for White and B/AA participants. For HC, significant proportional bias was observed across all devices in White participants (*P* < 0·010) but only for myBVI^®^ in B/AA participants (*P* = 0·024); however, coefficients for each device were similar between groups. There was no significant proportional bias observed for WHR estimates across devices, although the coefficients for WHR produced by ME360 were non-existent for White participants and high for B/AA participants.

## Discussion

While traditional tape measurements are considered to be cost effective and accessible, there are questions regarding their social acceptance and reliability^(24)^, particularly for those with overweight or obesity^(25)^. As such, this study sought comprehensively evaluate the precision and agreement of automated and clinically significant anthropometric variables across multiple mobile applications, and smartphones, against a reference tape measurement. The principle findings were (1) all variables produced by each mobile application and between smartphones exhibited acceptable precision which were comparable with tape measurements; (2) WC, HC and W:HT from all mobile applications demonstrated equivalence with tape measurements; however, WC was slightly underestimated, whereas HC was slightly overestimated; (3) the slight under- and overestimations for WC and HC were small enough to demonstrate equivalence, but resulted in non-equivalence for WHR across all automated methods; (4) there were no differences between WC produced by the automated methods and the reference in males, but WC was significantly underestimated across all applications in females and (5) some variation existed across applications, but all variables demonstrated slightly lower agreement for B/AA participants compared with White participants which may be a product of the weight status differences between groups or demographics of the populations used for method development. Overall, the results of our study support the use of WC and HC estimates produced from automated mobile applications, but demonstrates the importance of accurate automation for WC and HC estimates given their influence on other anthropometric assessments and clinical health markers.

First, each mobile application used in our study demonstrated acceptable precision for all automated assessments. There was a slight drop-off in precision when estimates were compared between smartphone types; however, precision remained within an acceptable range. While there are only a few studies that have evaluated the precision of automated anthropometrics using mobile applications^(16,26)^, there is contention regarding the precision of traditional tape measurements citing inaccuracies between self-measured and professionally measured assessments^(27)^ and measurements for those of higher weight status^(27,28)^. Another method shown to produce reliable digital anthropometrics is 3D optical scanning^(29)^. However, 3D scanners are generally unavailable for simple but important circumference measures in clinical practice, especially for those in lower socio-economic status or rural areas. Thus, the limitations of other methods highlight the need for precise estimates that are both accessible and cost effective. In our study, all automated anthropometrics produced precision estimates that were similar to the tape measurements performed in our study (which were conducted professionally) and those produced by 3D scanning in others^(9,29)^. Interestingly, the ME360_Apple_ application produced better precision estimates than those collected via tape measure with ME360_Samsung_ only marginally lower. It is possible that these small differences are due to differences in the developmental software (i.e. iOS or Android^®^), where initial mobile applications are built for a specific device (i.e. iPhone^®^ or Samsung^®^) with operating systems that require a particular coding language^(30)^. Despite this, all ICC were > 0·930 and PE were all ≤ 2·0 cm. Considering that this degree of precision was produced simply from two two-dimensional pictures on highly accessible mobile applications, it is plausible that these applications be considered reliable and comparable with traditional methods.

Several investigations have evaluated the agreement between automated anthropometrics from a mobile application and traditional tape measurements and have demonstrated considerable variation^(16,26,31,32)^, likely due to the different mobile applications employed in each study. Overall, WC, HC, and W:HT across all mobile applications in our study demonstrated equivalence to our reference method; however, there were slightly significant under- and overestimations for WC and HC, respectively, for each application. These bi-directional biases, where an underestimated WC was divided by a larger overestimated HC, resulted in significant underestimation of WHR for each application that did not demonstrate equivalence. So, while biases for WC and HC were relatively small, these small differences manifested in discrepancies across other variables. This relationship is also reflected in our results for W:HT, where all mobile applications underestimated W:HT as an extension of an underestimated WC. These small inconsistencies for WC and HC are problematic considering that WC and HC estimates are commonly used in other anthropometric screening tools to predict several health risks^(10,33)^. This is especially concerning considering the proportional biases and large LOA observed in our study. Specifically, we found that both ME360 applications demonstrated significant proportional bias for WC, where WC was underestimated to a greater degree in those with larger average WC. Significant proportional bias was observed for HC produced by both ME360 applications, but these biases were relatively small and much smaller than the proportional biases produced for WC using the same application. Because participants with a higher average WC had greater underestimations of WC without similar underestimations of HC, WHR was underestimated using this application. Interestingly, there was no proportional bias for WC using myBVI^®^; however, HC produced by myBVI^®^ demonstrated significant proportional bias, where HC was overestimated for those at larger average HC. Similar to ME360, albeit in differing directions, the overestimation of HC without simultaneous overestimations in WC led to underestimations of WHR. Therefore, while automated WC and HC estimates from a mobile application may demonstrate equivalence, those planning to employ these estimates as a part of a larger screening battery should do so with caution; although WC estimates produced by each mobile application did show utility in correctly determining abdominal obesity classification; a common cardiometabolic health risk assessment.

There are several issues that may explain our aforementioned results. First, the artificial intelligence used to develop each application is dependent upon the method used to train the application. For instance, if the mobile application was trained by a 3D scanner it is possible that comparisons to a tape measurement would result in lower levels of agreement. However, many mobile applications are trained by both 3D scanning and traditional tape measures, and recent investigations show agreement in body circumferences assessed by tape measurements, 3D scanners, and mobile applications^(16)^. Typically, these studies determine agreement by comparing automated measures to tape measurements taken at sites specifically defined by the mobile application^(16,31)^. While this methodological approach may result in better agreement, it may also limit real-world application given that self- or professionally measured circumferences may be taken from considerably different locations than from those suggested by the mobile application. Moreover, measurement sites may be markedly different between mobile applications making them difficult to compare. Therefore, to determine their ‘real-world’ performance we standardised the location of each tape measurement. It should be noted, however, that while the automated WC and HC were equivalent to tape measurements, this is specific to the performance of our investigators and the location in which our tape measurements were taken and thus, estimates taken by individual users, by other professionals, or at different locations may lead to differing results.

In addition to what has been previously suggested, our results may also be explained by our sex and race comparisons. For males, there were no significant differences between the WC measures and the reference for any mobile application. In fact, MD in WC for males were all ≤ −0·6 cm which is comparable to the results for both males and females produced by Nana et al.^(31)^ using a single mobile application. Conversely, WC was significantly underestimated by ≥ −3·0 cm across applications for females. Size differences between our female participants and those in the study by Nana et al.^(31)^ may explain the differences between studies, where our female participants had substantially higher weight, WC, and HC. However, male participants in our study were also much larger. As previously suggested by Neufeld et al.^(32)^, it is possible that differences in clothing between male and female participants, combined with the larger size of our participants, may explain our findings. Specifically, male participants wore only compression shorts/tights whereas female participants wore tights and a sports bra. It is common for females to wear high-waisted tights that cover a significant area (and often the majority) of the abdomen where the automated WC would be collected. Although steps were taken to alleviate this in our study, the automated landmarking procedures used by each mobile application may be altered when a considerable amount of abdominal mass is covered. Additionally, we observed that clothing worn by females was often tighter fitting than clothing worn by males. The tightness of the clothing, especially in female participants of larger body size, may alter the natural body shape in areas around the abdomen. It is also possible that the discrepancies in WC between the automated assessments and the reference in females was due to the inherent difficulty in measuring the female waist. As you descend the body’s vertical axis, the variation in WC along that axis is more exaggerated in females compared with males as a result of normal adult female body shape^(34,35)^. Thus, it is possible that these issues can introduce error into the automated measurement process as each application attempts to detect specific landmarks for its WC estimate.

The sex differences in HC for myBVI^®^ may also explain a number of our other findings. For males, myBVI^®^ significantly overestimated HC which was not observed for females. Interestingly, the majority of our male participants had either overweight or obesity, in addition to larger WC and HC, compared with our female participants. Considering that the proportional biases for HC from myBVI^®^ were nearly identical between sexes, it is more likely that the larger WC and weight status contributed to these differences. It is well known that males tend to deposit more fat in the anterior abdominal area whereas females tend to deposit more fat in the gluteal-femoral region^(36)^ leading to a larger WC for males. At higher degrees of overweight and obesity, the excessive accumulation of fat in the abdominal area is exposed to gravity and may extend well into the pubic region. Given that the pubic area is within the normal assessment region for HC, it is possible that the larger abdominal fat mass in male participants extended into the HC area for the automated assessments whereas tape measurements could avoid this interference. This potential discrepancy can also be observed in our comparisons by race. B/AA participants had significantly higher weight, WC, and HC compared with their White counterparts and 50 % of our B/AA participants had obesity, with all but one of our participants with severe obesity being B/AA males (the one participant was a B/AA female). Further, HC was overestimated to a greater degree for B/AA participants but without differences in proportional biases. Given the large differences in weight and WC and the lack of proportional bias in HC in this group, it is possible that at the extremes of abdominal adiposity, abdominal fat mass may impede the anterior area of the HC measurement resulting in an automated HC measurement that accounts for a portion of the waist, leading to HC overestimation. The potential impedance of the abdomen in larger individuals, coupled with the intrinsically larger distributions of fat in the gynoid region for B/AA individuals^(37)^, could result in larger measurement errors that may be exaggerated for B/AA males.

In conclusion, automated anthropometrics produced by two-dimensional pictures from mobile applications are cost effective and accessible tools that can be used to collect clinically significant anthropometric information. Automated anthropometrics from mobile applications demonstrate high levels of precision regardless of the smartphone used. Mobile anthropometrics also demonstrate agreement with traditional tape measurements (via equivalence testing) for WC and HC estimates. However, slight deviations in WC and HC, which may be due to technical issues that are exaggerated in estimates for female and B/AA participants, may lead to inaccuracies for other measurements such as WHR. As such, these data support the overall use of this technique for estimates of WC and HC, but individuals should include these data in comprehensive health assessments with caution given the range of individual error and over-and- underestimations. Despite this, WC estimates produced by each mobile application demonstrate utility in assessing abdominal obesity. These findings also support the potential use of this assessment method in prospective studies, where participants could self-assess across an intervention without the need for additional laboratory visits. However, future research examining the accuracy of self- and at-home assessments are necessary in addition to studies examining accurate assessment over time.
